# The impact of focused ion beam induced damage on scanning spreading resistance microscopy measurements

**DOI:** 10.1038/s41598-020-71826-w

**Published:** 2020-09-10

**Authors:** Komal Pandey, Kristof Paredis, Thomas Hantschel, Chris Drijbooms, Wilfried Vandervorst

**Affiliations:** 1 Imec, Kapeldreef 75, 3001 Leuven, Belgium; 2 Quantum Solid State Physics, KU Leuven, Celestijnenlaan 200D, 3001 Leuven, Belgium

**Keywords:** Characterization and analytical techniques, Microscopy, Techniques and instrumentation, Nanoscience and technology, Nanoscale devices, Electronic devices, Electronic devices

## Abstract

Scanning Spreading Resistance Microscopy is a well-established technique for obtaining quantitative two- and three-dimensional carrier profiles in semiconductor devices with sub-nm spatial resolution. However, for sub-100 nm devices, the use of focused ion beam becomes inevitable for exposing the region of interest on a sample cross section. In this work, we investigate the impact of the focused ion beam milling on spreading resistance analysis and we show that the electrical effect of the focused ion beam extends far beyond the amorphous region and depends on the dopant concentration, ion beam energy, impact angle, and current density. For example, for dopant concentrations between 1.0 × 10^20^ and 1.5 × 10^16^ cm^−3^ we observe dopant deactivation at least between 23 and 175 nm for a glancing 30 keV ion beam. Further, we show that dopant deactivation is caused by defect diffusion during milling and is not directly impacted by the presence of Gallium in the sample. Later, we also discuss potential ways to mitigate these effects.

## Introduction

As scaling progresses to sub-10 nm devices, 3D architectures, and complex integration schemes, the physical characterization of such devices is facing severe dimensional challenges. Among others, exposing the area of interest is a particularly important challenge for techniques targeting single device analysis. Due to its nano-meter scale precision, focused ion beam (FIB), especially Gallium (Ga)-based FIB, is extensively used for specimen preparation in transmission electron microscopy (TEM) and atom probe tomography. Moreover, the increasing demand for individual device analysis has expanded its use for preparing samples for other characterization techniques as well. For example, for scanning spreading resistance microscopy (SSRM) or scanning capacitance microscopy (SCM) measurements on cross-sections of sub-100 nm devices, the use of FIB has become indispensable. Additionally, the use of FIB becomes essential in the recently proposed hybrid characterization methodology whereby electrical scanning probe microscopy (E-SPM) measurements are conducted on a TEM lamella to combine structural, compositional and electrical studies on one single device^1^. During FIB sample preparation, the energetic Ga ions are incident onto the sample to induce physical sputtering of the material. The use of FIB, therefore, results concurrently in the implantation of Ga ions into the sample, which in turn, modifies its chemical composition, crystallinity and electrical properties. These structural and compositional changes are generally characterized using TEM and the studies in the past have shown that in case of silicon a 20–30 nm thick amorphous layer is formed after irradiation with 30 keV Ga ions^2–6^. The thickness can be reduced to 5–10 nm when using lower beam energies (5–10 keV)^7,8^. However, given that the sputtering rate decreases with ion beam energy and revealing the device of interest often requires the removal of hundreds of nanometres (or even microns), it is not always possible to operate at low beam energies. Furthermore, the reported reduction in amorphous layer thickness does not imply the complete elimination of the electrical damage, which is the main concern for electrical characterization techniques like SSRM, SCM etc. Hence, from an electrical characterization point of view, the use of FIB is not straightforward as its impact on the electrical properties of the device of interest may render the measurements inaccurate or, in the worst case, even worthless. Although depth profiles of Ga after FIB implantation have been reported in the past^9–12^, the information on its electrical impact is less detailed. So far only some authors^13–15^ have conducted studies on the lateral damage induced by FIB and concluded that indeed Ga irradiation modifies the electrical behaviour of the sample though without detailed data on the depth distribution of the electrical damage. With the introduction of 3D devices in IC industry, the knowledge of the complete 3D electrical damage imparted by FIB becomes very important while characterizing the devices electrically. In this work, we aim to gain insight on the extent of the FIB induced electrical damage in silicon and explore potential pathways to reduce this damage. To study the electrical impact of FIB, we employ-so-called scalpel SSRM^16^, a powerful method for controlled material removal that allows one to probe the third dimension.

## Method

**Figure 1:**
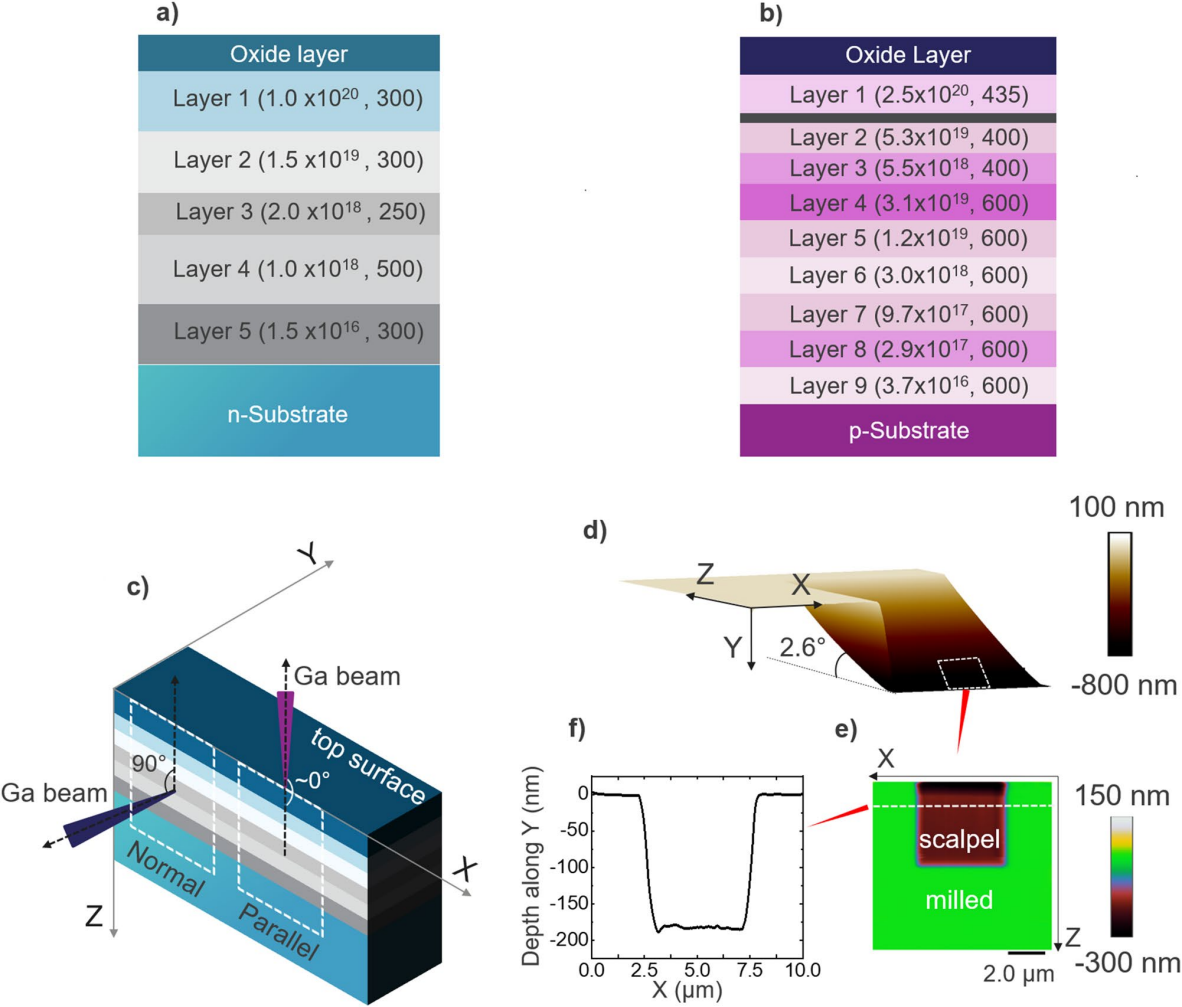
Details of sample preparation and scalpel method. Cross-sectional view of an (**a**) n-type (Arsenic doped) and (**b**) p-type (Boron doped) staircase sample. Corresponding doping levels (in cm^−3^) and layer thicknesses (in nm) are indicated within brackets. (**c**) Schematic of the staircase sample with parallel (case Parallel) and normal incidence (case Normal) of Ga beam on the sample cross-section. (**d**) 3D view of an AFM topography image seen after FIB milling (parallel incidence). Scalpel SSRM was performed within this FIB milled region. (**e**) Crater depth read out using AFM after the scalpel SSRM (brown region) on a FIB milled region (green area). (**f**) A line scan taken along the dashed line through the scalpel area.

All the measurements were performed on dedicated in-house staircase samples^17,18^, as schematically shown in Fig. 1a,b for n-type (CS01-SiAs) and p-type (CS08-SiB) respectively. The samples contain multiple uniformly doped epitaxially grown silicon layers covering the doping range from 1.5 × 10^16^ to 2.5 × 10^20^ cm^−3^. The doping level (in cm^−3^) and thickness (in nm) of each layer are indicated in Fig. 1a,b. Since SSRM measurements were carried out on the sample cross-section, an additional 400 nm oxide layer was deposited before the measurements to protect the edge of the sample cross-section.

All the FIB related experiments were conducted using a FEI Helios450 dual-beam FIB/SEM. Figure 1c illustrates the two different FIB milling configurations discussed in this paper. The Ga ion beam was directed either parallel (case Parallel termed hereafter) or normal (case Normal) to the sample cross-section. Large rectangular regions were milled on the sample cross-section using ion beam energies between 5 and 30 keV, and a current ranging from 80 pA to 22 nA. For normal incidence of the Ga beam, the dimensions of these regions were kept 100 µm in X direction and at least 20 µm in Z direction. Since the size control of the milling region along Z direction is difficult in case Parallel, unless stated otherwise, we made sure that all doped layers receive a high enough Ga dose to reach the steady state condition between implantation and sputtering i.e. the sputtered material thickness is at least equal to the sum of the projected peak Ga range and straggling (R_P_ + σ).

To reveal the post milling topography for case Parallel, we have imaged the edge of the crater with atomic force microscopy (AFM) as shown in Fig. 1d. Compared to the pristine surface the crater is formed extending roughly 20 µm into the substrate (Z-direction) at a constant slope of ~ 2.6° with a maximum depth of 800 nm. This indicates that the Ga beam is not completely parallel to the sample cross-section (XZ plane). After FIB processing, scalpel SSRM was used for the characterization of the electrical damage depth distribution inside the milled area, which is typically 5 × 5 µm^2^ starting at the sample edge. For all the measurements reported in this paper, the depth during the scalpel process is defined as the distance perpendicular to the original FIB milled area (~ Y-direction). This is clarified in Fig. 1d–f, where we show AFM images of the FIB milled area before and after performing scalpel SSRM.

Scalpel SSRM is a 3D extension of SSRM, a well-established technique for 2D carrier profiling of semiconductor devices^18–23^. Fundamentally, SSRM is an electrical AFM based technique in which a highly conductive diamond tip is scanned over the sample surface at high force (in the order of a few µN). The high force is used to lower the tip-sample contact resistance by inducing the so-called β-Sn phase transformation occurring at a pressure of about 8–12 GPa underneath the tip and to maintain a good electrical contact between them^24^. Applying a constant bias between the tip and the sample back contact allows us to probe the spreading resistance, which, in most cases, dominates all other resistances present in the system. According to Eq. (1), the spreading resistance (R_spreading_) scales linearly with sample resistivity (ρ_sample_), where “a” is the electrical contact radius. However, as the contact radius is hard to determine experimentally, the direct calculation of resistivity (hence the carrier concentration) is impossible. Therefore, we rely on the use of a calibration standard to convert the measured spreading resistance to resistivity or carrier concentration. Further information on SSRM can be found in the literature^18^.

1 $${R}_{spreading}=\frac{{\rho }_{sample}}{4a}$$

As the force induced contact is the key to the SSRM measurements, tip induced material removal is inevitable. In scalpel SSRM, we exploit this property to reach a controlled material removal whereby the degree of removal is dependent on the applied force on the tip. In this manner, a series of consecutive 2D resistance maps is obtained, each corresponding to a different depth. It has been established that the layer removal can be controlled down to a few nm leading to scalpel based SSRM depth profiles with similar depth resolution^25^. When applied to the FIB milled regions, the information on the FIB-related 3D-electrical damage can be obtained by acquiring such 2D-resistance maps and comparing them to similar maps obtained on pristine regions. All SSRM (and scalpel SSRM) measurements reported in this paper were carried out on a Bruker Dimension ICON AFM equipped with an SSRM module. We used imec boron doped full diamond tips (FDT)^20^ with an average spring constant of 27 N/m. These tips are extremely hard and wear resistant and enable us to perform reliable electrical measurements during the entire scalpel run. To avoid the effect of tip variations in the SSRM measurements, the same probe was used throughout a single scalpel run and all the measurement parameters were kept constant. Note that we carried out reference measurements on the pristine region before and after the scalpel run to exclude any impact of tip degradation on our results. Only the scalpel runs without a significant difference (< 25%) between these two measurements were included in the analysis. In this work, a layer in the irradiated area is considered electrically damaged if its measured resistance is 25% higher (which corresponds to the typical error in SSRM resistance measurements) than the resistance of the corresponding layer in the pristine area. For each layer, the measured SSRM resistance in the pristine region will be referred as the ‘expected resistance’ for that layer. To ensure an accurate estimate of the depth of each resistance scan, AFM topography measurements were carried out after every 2–3 SSRM scans and an average removal rate per scan is obtained. An example of the crater depth read out during scalpel SSRM is shown in Fig. 1e,f. During all SSRM measurements, a sample bias of − 0.5 V (and 0.5 V) was applied for n-type (and p-type) while the tip was grounded. The uniformity of the scanned area is ensured by keeping the scan line density sufficiently large, i.e. above 0.1 line/nm^26^.

## Results

**Figure 2:**
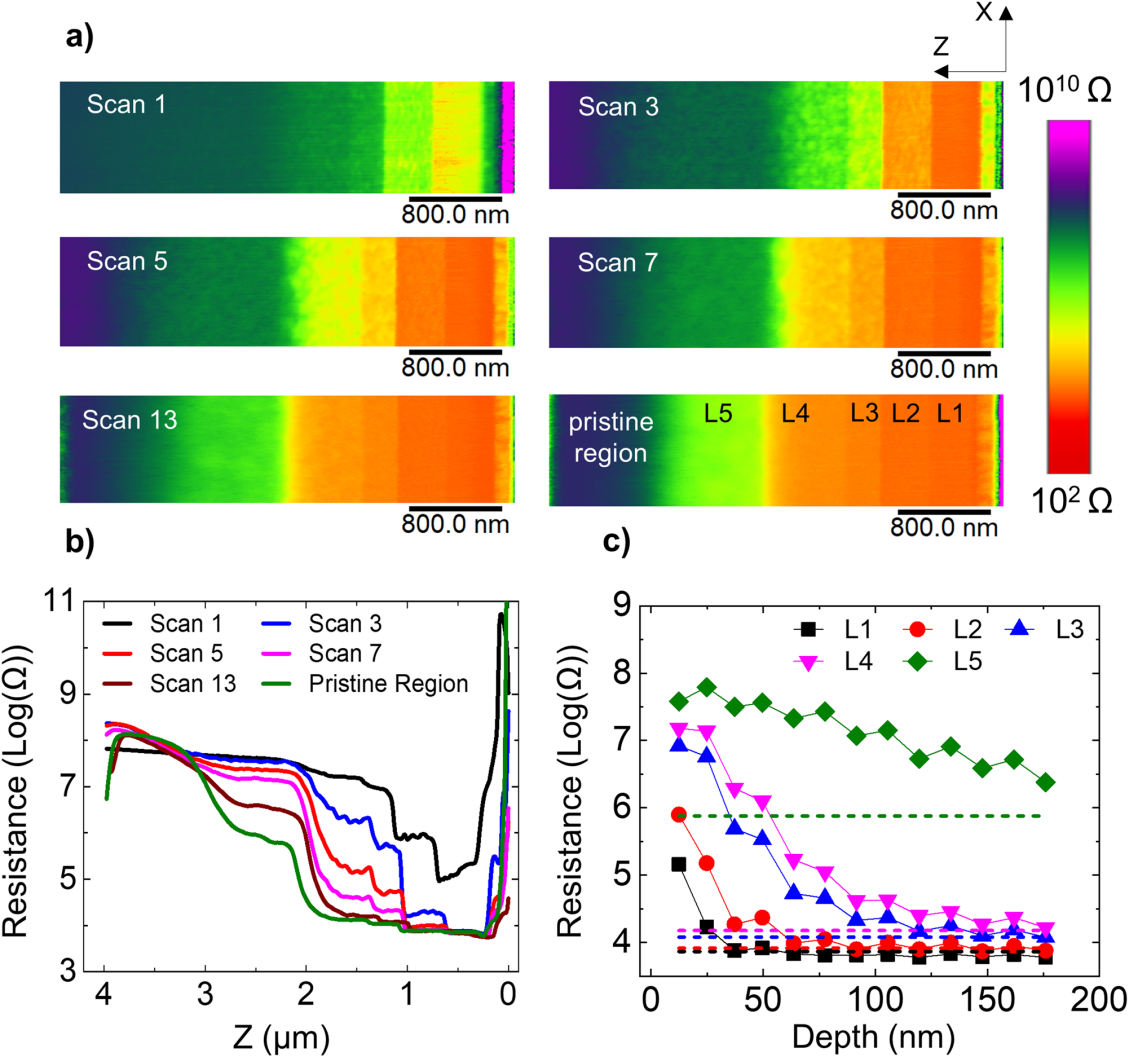
Details of scalpel SSRM on a FIB milled area of the n-type staircase. The FIB milling was performed according to case Parallel with 30 keV beam energy. (**a**) 2D-resistance maps (in XZ plane) across milled region. Increasing scan numbers reflect scans at increasing depths along Y direction. A 2D resistance map of the pristine region is added for comparison. (**b**) Resistance value versus Z-direction extracted from lines scans through (**a**). (**c**) The evolution of resistance value along the scalpel depth. Dashed lines indicate the resistance values obtained on different layers (coloured accordingly) within the pristine region.

To study the impact of the Ga FIB beam on the electrical properties of Si, we have performed scalpel SSRM on a parallel milled area on the n-type staircase. Figure 2a represents the 2D SSRM resistance maps (covering the XZ plane in Fig. 1c) obtained at successive depths. For comparison, a resistance map taken on an area, which was not subjected to FIB milling i.e. pristine region, is also shown. Note that the pristine area is chosen more than 500 µm away from the FIB milled area to avoid any contamination or damage originating from the ion beam tails. From the first scan taken at the surface of the milled area, it is clear that the measured resistances of all five layers are significantly higher than their expected values. Interestingly, the relative order of the doping levels is preserved, i.e. the highest dopant concentration shows the lowest resistance and vice versa. Moreover, the difference between the resistance levels of consecutive highly doped layers (> 10^18^ cm^−3^) is larger than the corresponding differences on the pristine surface, indicating an increased sensitivity in the first few scans for these doping levels. The lower doped layers, however, can hardly be distinguished and the sensitivity is lost. Although after the 3rd scan (~ 37 nm removed) all five layers are again qualitatively distinguishable, some layers still show an increased resistance. Figure 2b shows the averaged resistance profiles along Z of each resistance map in Fig. 2a. It is evident here that with increasing SSRM scans (i.e. material removal) the resistance of each layer progressively decreases towards its expected value i.e. the resistance value obtained from a pristine region. By converting the scan number to removed depth, we plot the evolution of the resistance as a function of depth (into the milled area), as shown in Fig. 2c. Each data point in the curve represents the average resistance measured in a layer and the dashed lines represent the expected resistances of the layers as observed in the pristine region. These results show the extent of the electrical damage of FIB. A strong dependence of the electrical damage on the carrier concentration is observed. For example, in the highest doped layer the expected resistance value is already reached after removing only 37 nm, whereas for the lowest doped layer the impact is observed even at a depth of 175 nm. Note that we observe a small oscillation in the data, primarily in the lowest doped layer, where consecutive data points correspond to opposite slow scan directions (X and − X). As the tip moves, the effective tip-sample contact may slightly differ in both directions due to small tip asymmetries, and hence results in slightly different resistance values*.*

From these observations, we can state that FIB milling causes an increase in the resistance of all the five layers, irrespective of their doping concentrations, be it that for higher doping levels the effect disappears at shallower depths as compared to the case of lower doping levels. In principle, SSRM measures the total resistance of the system, which, in ideal measurement conditions, is dominated by the spreading resistance. In the present experiment, however, two main sources for the overall increased resistance can be listed: (1) the presence of an additional series resistance originating from a lower mobility region in the milled area, and dominating the spreading resistance, (2) the loss of active carriers in the sample. The interaction of the Ga ion beam with the sample during FIB milling involves several phenomena that can lead to these effects. First, the formation of an amorphous layer (total loss of crystallinity) results in the reduction of the carrier mobility in the affected layer leading to a local increase in the resistivity. Second, substitutional dopants can be knocked out from their lattice sites as a result of the atomic collisions occurring during the interaction of the Ga-atoms with the silicon lattice. This induces deactivation of the dopants and thus an increase of the resistivity. Third, during the atomic collisions, the incoming Ga ion transfers a part of its energy to the silicon lattice atoms. If the transferred energy is greater than the displacement energy of the silicon atom in the lattice, the silicon atom will be ejected from its lattice site, creating a vacancy-interstitial pair. These vacancies and interstitials are known to form complexes with dopant atoms^27–29^. Hence, their formation can play a role in deactivating dopants and consequently, in increasing the resistivity. Finally, the incorporation of Ga atoms, which, if electrically active, act as p-type dopant in silicon and counteract (enhance) the original n-type (p-type) doping. In this case, the incorporation of a certain concentration of active Ga dopants will be most apparent for layers with low doping concentrations. For layers with very high doping concentrations, the observable counter (or enhanced in p-type) doping from the active Ga dopants would be negligible. However, for low doping concentrations, and depending on the dopant type (n or p), the Ga incorporation will lead to a higher (or lower) spreading resistance values for n-type (p-type) material. Our definition of “electrical damage” refers to the combined result of all these phenomena.

**Figure 3:**
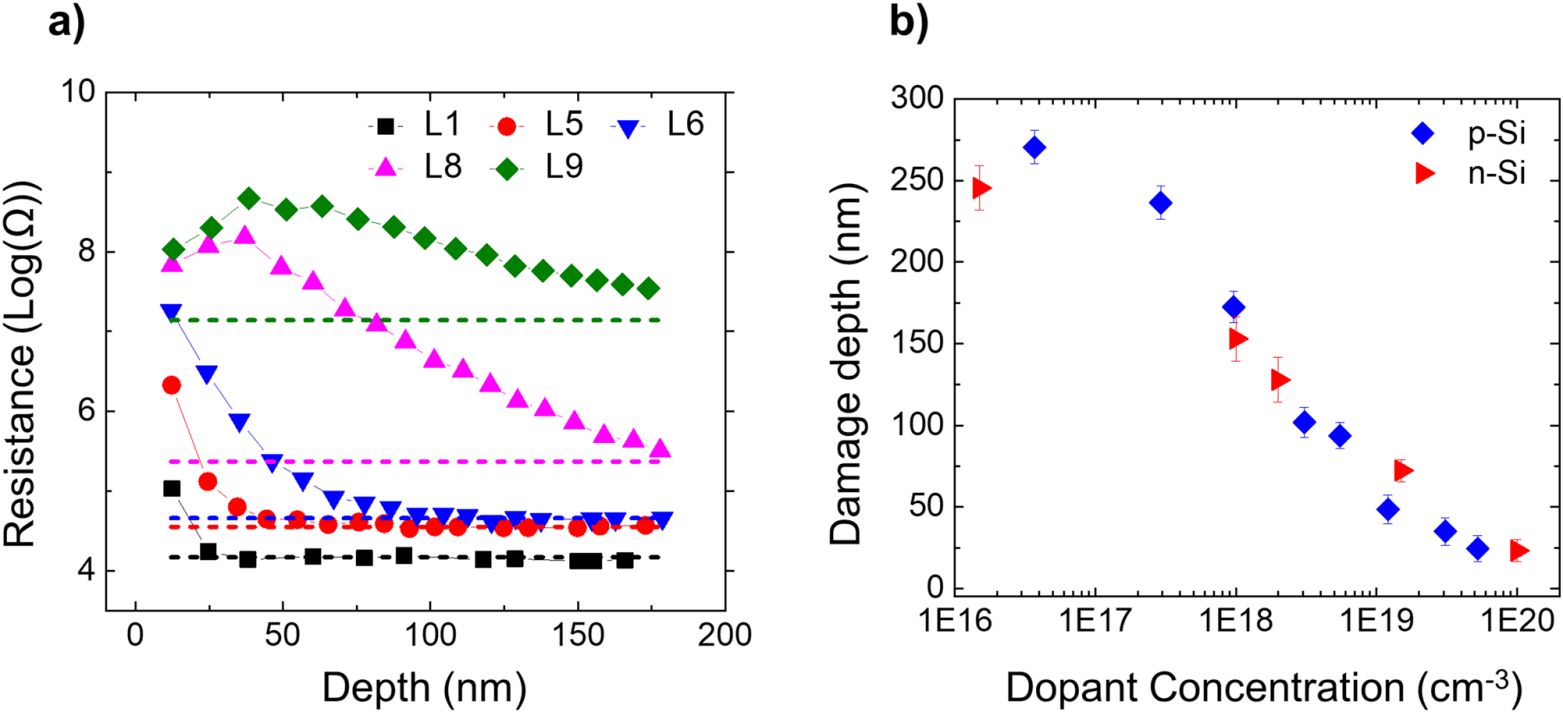
A comparison of damage depth in n- and p-doped samples is made. (**a**) Resistance depth profiles measured on an area irradiated with 30 keV of Ga beam, incident parallel to the cross-section of p-type Silicon staircase sample. (**b**) The electrical damage depths as a function of initial carrier concentration of stair-case layers for both p-Si and n-Si.

To explore the possibility of the incorporated Ga ions being active, an identical experiment was performed on a p-doped silicon staircase sample (Fig. 1b) whereby the FIB processing parameters were kept the same as for the previous measurements shown above for n-type (Fig. 2). The results, shown in Fig. 3a, indicate that, like n-type, all p-type layers show an overall increase in their resistance values. This identical behaviour regardless of the dopant type rules out the possibility that implanted Ga atoms act as substitutional dopant atoms and influence the observed spreading resistances. From the resistance-depth curves (Figs. 2c and 3a), we can obtain the extent of the electrical damage for all carrier concentrations (both n- and p-type), as shown in Fig. 3b. The electrical damage depth is determined for each doping layer as the depth where the resistance reaches the pristine level within 25%. In other words, beyond that depth, the impact of the electrical damage is no longer significant. By combining all these initial concentrations and depths, we obtain the observed depth of the electrical damage as a function of the initial dopant concentration, as shown in Fig. 3b. As no significant difference is observed between n-type and p-type, we will focus in the next sections of this paper only on the n-type sample. Note that we take 25% of the total measured resistance value to determine the threshold for the electrically damaged depth. In the lower doped regions, the measured resistance is dominated by the spreading resistance, while in the highly doped regions, the tip resistance can be of the same order of magnitude as the spreading resistance. Therefore, adding the tip resistance in our 25% thresholding ultimately leads to an underestimation of the damage within the highest doped layer. For instance, from Fig. 2, the tip resistance can be estimated as 4,570 Ω, which is approximately 2.2 times of the spreading resistance in the highest doped layer. Therefore, taking 25% of the total resistance rather than the spreading resistance leads to an underestimation of around 8 nm, which is within the error of our measurements.

Another observation here is that the electrical damage observed in SSRM extends much further (up to 300 nm) than the reported thickness of the FIB induced amorphous layers in silicon, which is only 20–30 nm^2–6^. Therefore, the electrical damage sensed by SSRM cannot be solely attributed to the presence of an amorphous layer. This is not very surprising as this amorphous layer only reflects the region where the Ga induced crystal damage leads to a total loss in crystallinity and does not represent the maximum depth up to which Ga ions penetrate into the solid. Beyond the amorphous layer, the Ga ions still induce individual collision cascades and atom displacements, which could give rise to dopant and substrate atom displacements and reduced mobility. However, a strong dependence of the electrical damage depth on the initial doping level suggests that the effect is not solely caused by the reduction in mobility. For instance, L1 and L2 have very similar mobility values, yet show a strong dopant dependent difference in damage depth, indicating that mobility degradation is not the dominant mechanism. Hence, the two important questions that arise from these observations are: (1) What is the main reason for such a large extent of electrical damage? and (2) What is the relation between the incorporated Ga depth profile and the extent of the electrical damage? For this purpose, a detailed study is conducted within which the effect of various FIB milling parameters on the electrical damage is analysed.

### Effect of ion dose

**Figure 4:**
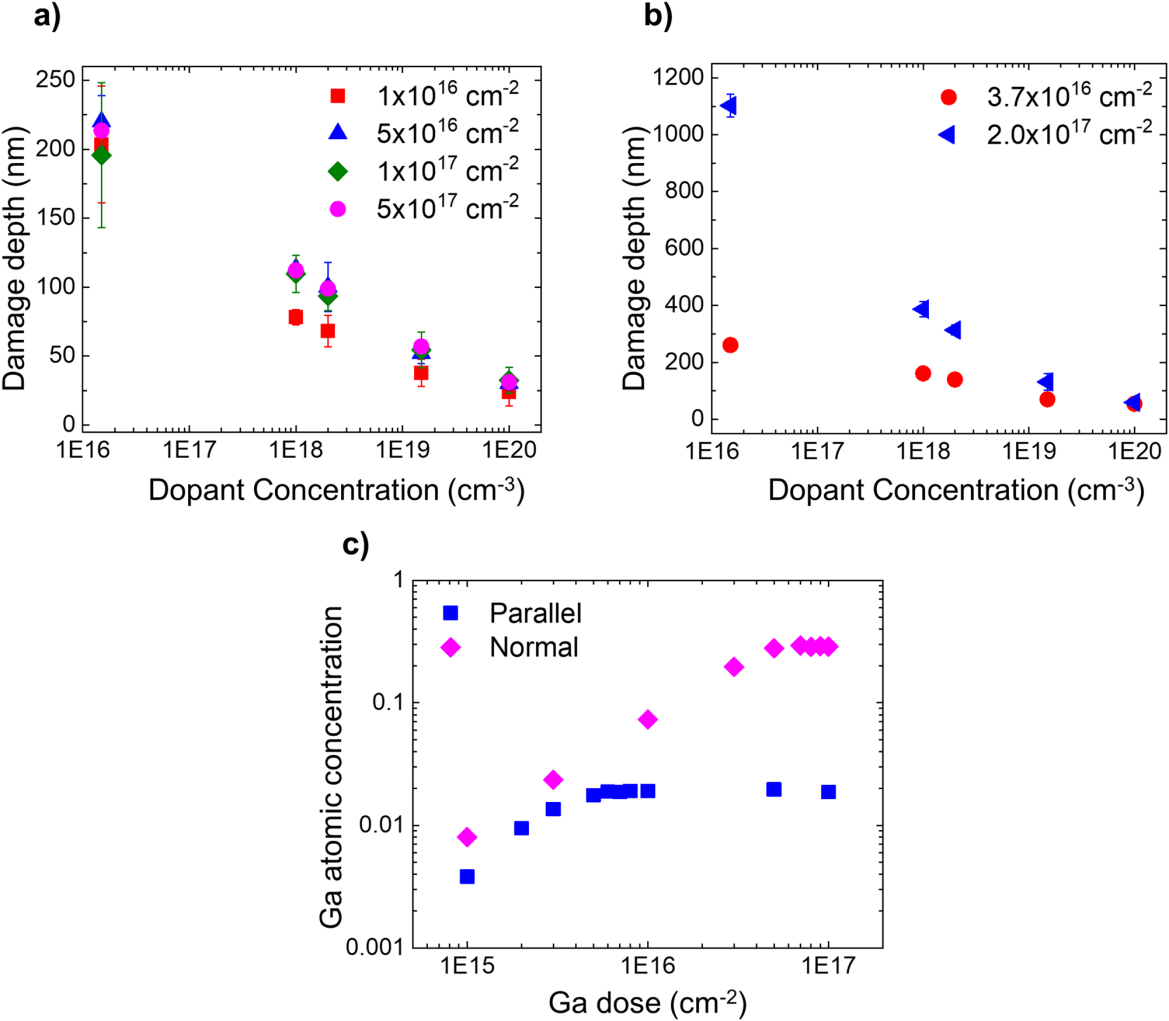
The impact of Ga ion dose on SSRM measurements is studied for two incident beam angles. The electrical damage depths are plotted as a function of doping concentrations for various Ga doses used during FIB milling for (**a**) parallel and (**b**) normal beam incidence angles. The beam energy used was 30 keV. (**c**) The calculated peak Ga concentration is plotted as a function of incident Ga dose for case Parallel (blue squares) and case Normal (pink diamonds). These results illustrate the different Ga dose required to reach steady state in case Parallel and case Normal.

Rectangular areas are milled at a beam incidence angle of 3° (equivalent to case Parallel) using a 30 keV beam energy. The ion dose is varied from 1.0 × 10^16^ cm^−2^ to 5.0 × 10^17^ cm^−2^ using a beam current of 2.5 nA. Figure 4a represents the electrical damage recorded as a function of the doping concentration of the layers for various implanted doses. It is clear from the graph that increasing the ion dose beyond 5.0 × 10^16^ cm^−2^ does not increase the damage in the sample, implying that there exists a critical dose beyond which the electrical damage in the sample does not increase further. Since we saw a small increase in damage depth by increasing the dose from 1.0 × 10^16^ cm^−2^ to 5.0 × 10^16^ cm^−2^ for case Parallel, it can be concluded that the steady state dose lies somewhere between these two values. Whereas, in case Normal (see Fig. 4b), still a significant increase in the damage depth is observed when the dose was increased from 3.7 × 10^16^ cm^−2^ to 2.0 × 10^17^ cm^−2^.

These data indicate that the steady state dose is reached much earlier in case Parallel as compared to case Normal. The latter can be explained by realizing that during ion implantation, a steady state in terms of ion incorporation is reached when any additional dose does not increase the ion content in the sample as its increase is counteracted by the sputtering process^30^. When reaching this steady state, the concentration of the implanted species reaches its maximum value. Using the SDTrimSP software^31^ (version 6.01) in dynamic mode, we calculated the maximum Ga concentration incorporated in Si as a function of implanted dose for both case Parallel and case Normal. The result of these calculations is shown in Fig. 4c, where it is evident that the steady state condition in case Parallel is reached at a much lower dose (8.0 × 10^15^ cm^−2^ vs. 9.0 × 10^16^ cm^−2^) as compared to in case Normal. As discussed in reference^30^, the steady state dose equals to $\frac{{n}_{0}{R}_{p }\alpha }{Y}$, where n_0_, R_p_, α and Y are the atomic density of the target material, mean range of the ions into the target, accommodation coefficient and sputter yield respectively. This implies that a higher sputter yield will lead to a smaller steady state dose and a lower accommodation coefficient will result in a lower Ga incorporation. In view of the angular dependence, the ratio of accommodation coefficient to the sputter yield i.e. $\frac{\alpha }{Y}$, is nearly 15 times lower in case Parallel as compared to case Normal. Therefore, in case Parallel the steady state dose is expected to be nearly 15 times lower than in case Normal, which is in line with the data in Fig. 4c. In addition to this, we notice that the steady state dose in terms of electrical damage is nearly the same as the steady state dose for Ga implantation. This implies that once the steady state is reached in terms of Ga incorporation, further bombardment does not increase the number of both Ga ions and the defects that are responsible for electrically damaging the sample. Hence, also here a dynamic equilibrium between Ga incorporation and/or defect injection and material removal (with its associated Ga and defect content) is established. Any further increase in the Ga dose does not increase the concentration of defects but leads to further material removed from the surface, and thus shifts the damaged region deeper into the sample.

As the use of FIB in SSRM is to mill the surface such that the device of interest can be exposed for analysis, it is fair to assume that the stationary state is always reached. Thus, we limit our study to doses higher than the corresponding steady state doses in both cases (Parallel and Normal).

### Effect of beam incidence angle

**Figure 5:**
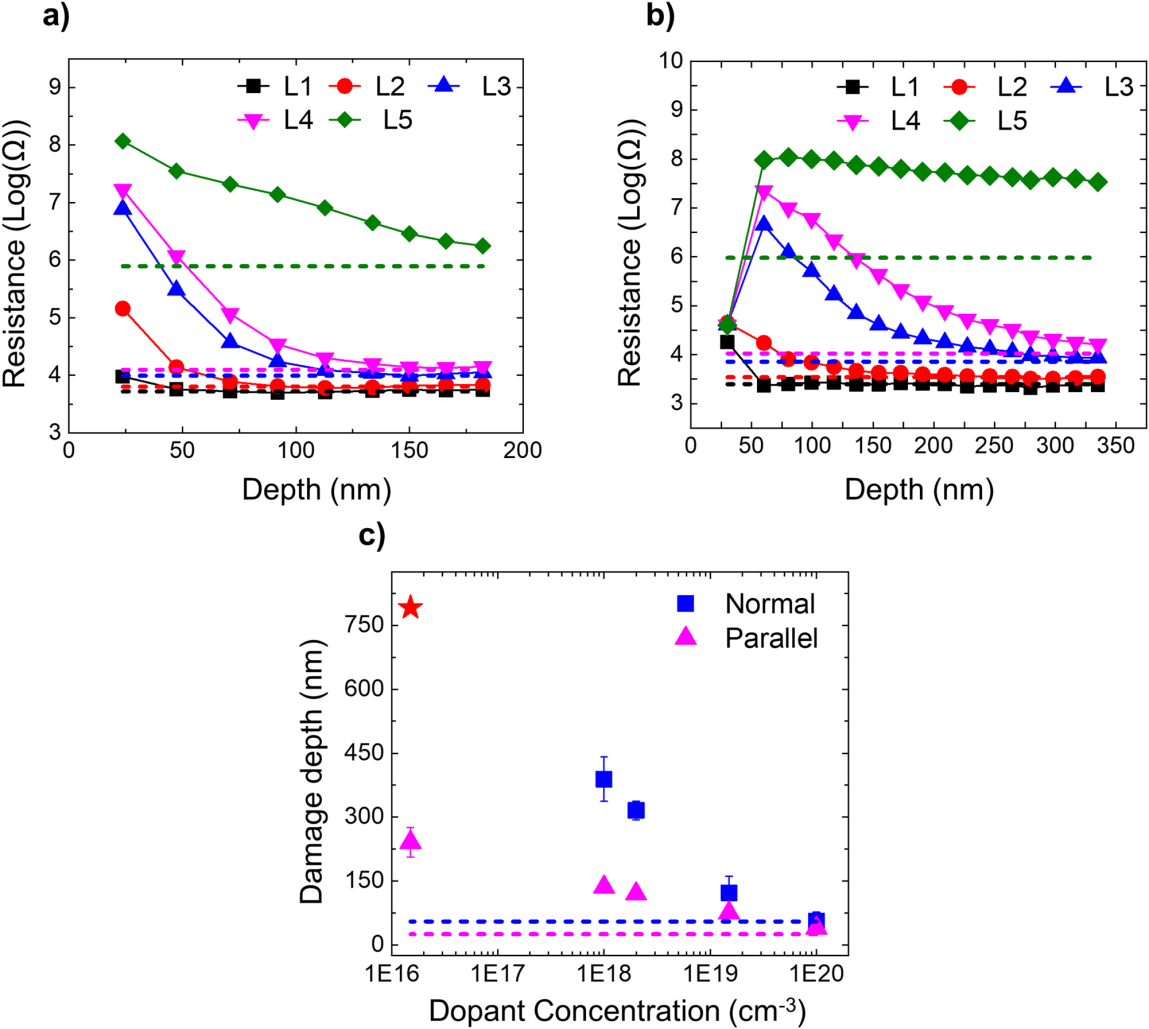
The impact of beam incidence angle on SSRM measurements is studied using scalpel SSRM. Resistance depth profiles measured on an area irradiated at 30 keV Ga beam energy, incident (**a**) parallel and (**b**) normal to the cross-section of n-type silicon staircase sample. (**c**) The measured electrical damage depths as a function of initial carrier concentration of differently doped layers are plotted for case Parallel (pink triangles) and case Normal (blue squares). The extents of the amorphous regions are indicated by the dashed pink (case Parallel) and blue (case Normal) lines.

**Figure 6:**
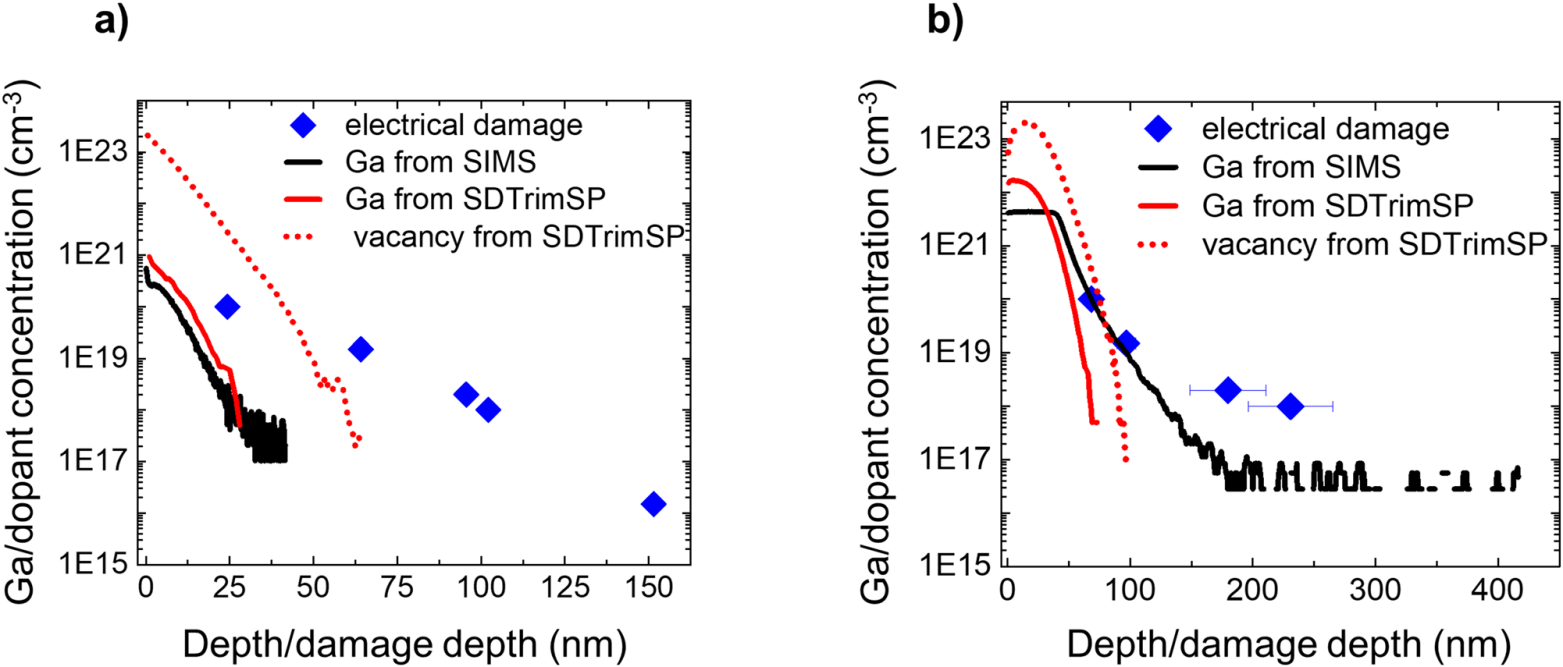
The effect of Ga penetration depth on electrical damage is studied using SIMS. Ga depth profiles (solid black) obtained from SIMS measurements for 30 keV Ga beam are shown for (**a**) parallel and (**b**) normal incidence of Ga beam. The Ga (solid red) profiles as calculated from SDTrimSP software (version 6.01) are also plotted for comparison. The simulated vacancy profiles (red dots) in both cases (i.e. case parallel and case Normal) follow corresponding Ga profiles. The electrical damage depths (blue diamonds), shown for various doping concentrations, represent the depth beyond which the effect of FIB milling is insignificant in SSRM measurements.

As indicated in the previous paragraph, the angle of incidence does play an important role in terms of Ga incorporation (depth range, steady state maximum concentration and steady state dose). To investigate their impact more extensively, we studied the effect of the two different angles of incidence (i.e. case Parallel and case Normal) in more detail. Figure 5a,b represent the resistance-depth profiles obtained after milling two areas according to case Parallel and case Normal respectively, with a beam energy of 30 keV and a beam current of 80 pA. We used a dose of 1.0 × 10^17^ cm^−2^, which is higher than the steady state dose in both cases. In both cases, all the layers first show a high resistance value which decays slowly towards their expected resistance values (pristine values), which are indicated by dashed lines. A comparison of the electrical damage depths for case Parallel and case Normal, as shown in Fig. 5c, reveals that the difference in the Ga incidence angle leads to a significant difference in the electrical damage as measured by SSRM. Particularly, in the low concentration regime (< 10^18^ cm^−3^) the electrical damage depth in case Normal is almost 3 times larger than in case Parallel. Note that we could not reach a damage free depth for the lowest doped layer i.e. 1.5 × 10^16^ cm^−3^ (see Fig. 5a,b) due to the tip degradation during the scalpel process (the material removal in scalpel SSRM is therefore limited to ~ 500 nm, in depth). The corresponding data point in Fig. 5c (as indicated in red star) is estimated by an exponential extrapolation of the data obtained. Interestingly, in both cases the electrical damage depths measured in the highest doped layer i.e. 1 × 10^20^ cm^−3^ reflect the corresponding thicknesses of amorphous layer. The latter are indicated by dashed lines (coloured accordingly) in Fig. 5c and amount to ~ 25 nm for case Parallel and ~ 55 nm for case Normal^4–6,32^. Since the Ga penetration depth is reduced by reducing the incidence angle (parallel vs. normal), one could be tempted to relate the difference in the electrical damage between case Normal and case Parallel to the different extent of the Ga penetration in the sample. To elucidate this potential correlation, the concentration-depth profiles of the Ga ions are measured using secondary ion mass spectrometry (SIMS) for the two FIB-geometries. The SIMS results are shown in Fig. 6 together with the Ga and vacancy depth profiles calculated from Monte Carlo simulations using SDTrimSP software^31^ (version 6.01) and the electrical damage depths for various doping concentrations.

In both cases, the Ga penetration depth is substantially smaller than the corresponding extents of the observed electrical damage. In other words, for a layer the electrical damage is still present at a depth at which the Ga concentration is below the doping concentration of the layer. Thus, no direct correlation between the Ga-penetration depth and the extent of electrical damage depth can be established, implying that the physical presence of Ga in the sample is not directly responsible for the observed impact on the electrical measurements. Similarly, as the vacancy profiles exhibit a similar shallow distribution, no direct link to the damage can be made. Nevertheless, some correlation must be present as we did observe that the electrical damage distribution is reduced by the angle of incidence (see Fig. 5c) which also reduces the Ga-incorporation (and vacancy) depth.

In that respect, we must note that these calculations ignore any further in-diffusion of the vacancies (or Ga) during the Ga-bombardment, while room temperature diffusion of point defects is known to occur over large distances^33^. This means that the deeper electrical damage observed in our case could be a result of this in-diffusion and a coupling between dopant deactivation and defect density. If the latter were correct, an estimate of the relative importance of this process can be obtained by considering the total amount of injected defects for case Parallel and case Normal. By taking the integral of the simulated vacancy profiles, as shown in Fig. 6, an approximately 4.5 times higher defect density is found in case Normal than in case Parallel, which could explain the observed differences seen in their electrical damage distributions.

Furthermore, in both cases we notice that the Ga tail goes deeper than predicted by the Monte Carlo calculations. Such deep Ga tails could be the result of either Ga ion channelling or Ga in-diffusion. Although these calculations do not account for ion channelling, we must note that channelling prevails at lower dose when the lattice is free from defects. At high dose, the amorphous layer^34,35^ significantly reduces the channelling probability. Since the dose used in both experiments exceeds the steady state dose, and thus sputtering occurs, it is reasonable to assume that the initial channelling tail is removed. This implies that Ga in-diffusion is mainly responsible for producing these tails. As Ga diffusion in silicon is reported to happen through vacancy mechanism^36,37^, this indicates that defects are indeed mobile and that their in-diffusion may be responsible for the large electrical damage depths.

### Effect of current density

As suggested from the results so far, the diffusion of point defects is expected to be the ultimate process governing the damage formation during FIB irradiation. Since diffusion is a time dependent phenomenon, the duration between two consecutive interactions between an incoming ion and a silicon atom (i.e. the current density) will affect the diffusion (and/or annihilation) of defects, similar to the impact of the current density on the amorphous layer thickness^38^.

**Figure 7:**
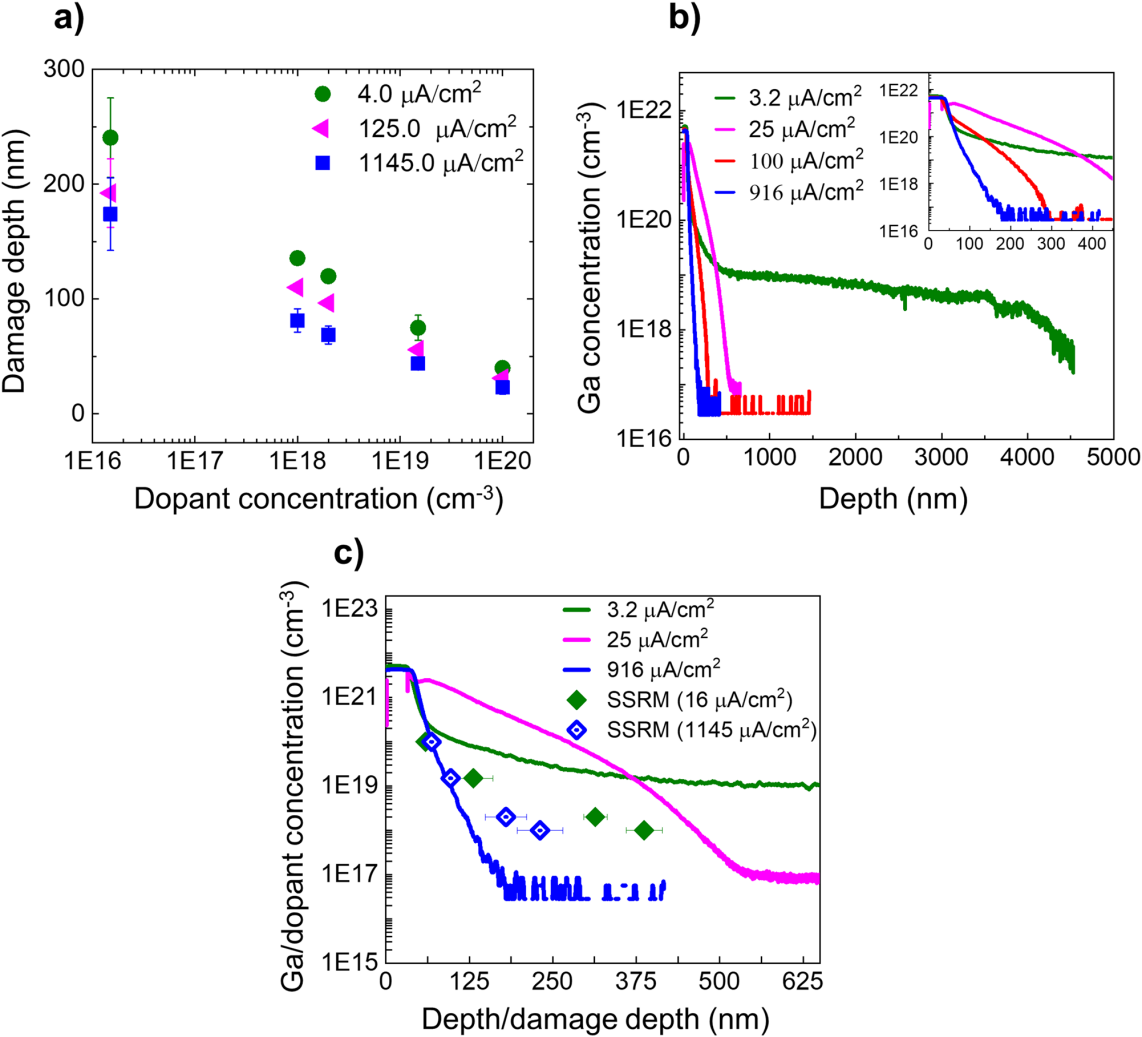
The effect of current density on the electrical damage depth is studied using the scalpel method. (**a**) The electrical damage depth as a function of doping concentration is plotted for various current densities for a 30 keV ion beam parallel to the n-type staircase sample. (**b**) The Ga profiles for various current densities are measured using SIMS on an area which was irradiated at normal incidence. A zoom of the profile is shown in the inset to highlight the severity of the Ga in-diffusion. (**c**) The Ga profiles for case Normal are compared with the corresponding electrical damage depths measured by SSRM.

It is, therefore, worthwhile to investigate the effect of current density, which determines the rate of incident ions within an area. For this purpose, we varied the beam current from 80 pA to 22 nA on an area of 20 × 100 µm^2^, while keeping the scan speed constant. The incident dose was kept at 1.0 × 10^17^ cm^−2^ using a beam energy of 30 keV at parallel incidence. The corresponding electrical damage depths for various doping concentrations are shown in Fig. 7a. It is evident that increasing the current density reduces the overall damaged region. Although the effect only leads to a 40% reduction in electrical damage depth by increasing the current density by nearly 2.5 orders of magnitude, it does provide further evidence that defect injection, diffusion and annihilation contribute to the extent of the electrical damage.

In the discussion on Fig. 6, we showed that there was no direct correlation between the Ga penetration and electrical damage depths, but one could argue that for case Normal the electrical damage distribution seems to follow the Ga tail. This similarity is purely coincidental as evidenced by observing the Ga profiles and the damage depth distributions over a wider range of conditions. When considering the Ga profiles (Fig. 7b) for case Normal, the Ga profiles depend dramatically on the current density and indicate a very strong in-diffusion at low current densities (particularly between 3.2 µA/cm^−2^ and 25 µA/cm^−2^), while the damage depth variation is limited (see Fig. 7c for case Normal). Although the SSRM data were not taken at the exact same current densities that were used to measure Ga depth profiles, it is clear from Fig. 7b that the Ga depth profile at 1,145 µA/cm^−2^ would be shallower than the Ga profile shown at 916 µA/cm^−2^. This means that the electrical damage at 1,145 µA/cm^−2^ is deeper than the corresponding Ga profile. Whereas at the current density of 16 µA/cm^2^, the Ga profile (located somewhere between the one for 3.2 and 25 µA/cm^2^) would be deeper than the corresponding electrical damage depths. This confirms that there is no simple relation between the Ga profiles and the SSRM electrical damage. For example, when considering the electrical damage depth for a dopant concentration of ~ 1.5 × 10^19^ cm^−3^ (at a current density of 16 µA/cm^−2^), the corresponding Ga concentration exceeds the initial dopant concentration by at least a factor of 10, again emphasizing its negligible effect on the SSRM results. On the other hand, the very large Ga in-diffusion does suggest that a similar diffusion of point defects can occur, thereby, leading to the large electrical damage depths as observed in SSRM.

### Effect of beam energy

**Figure 8:**
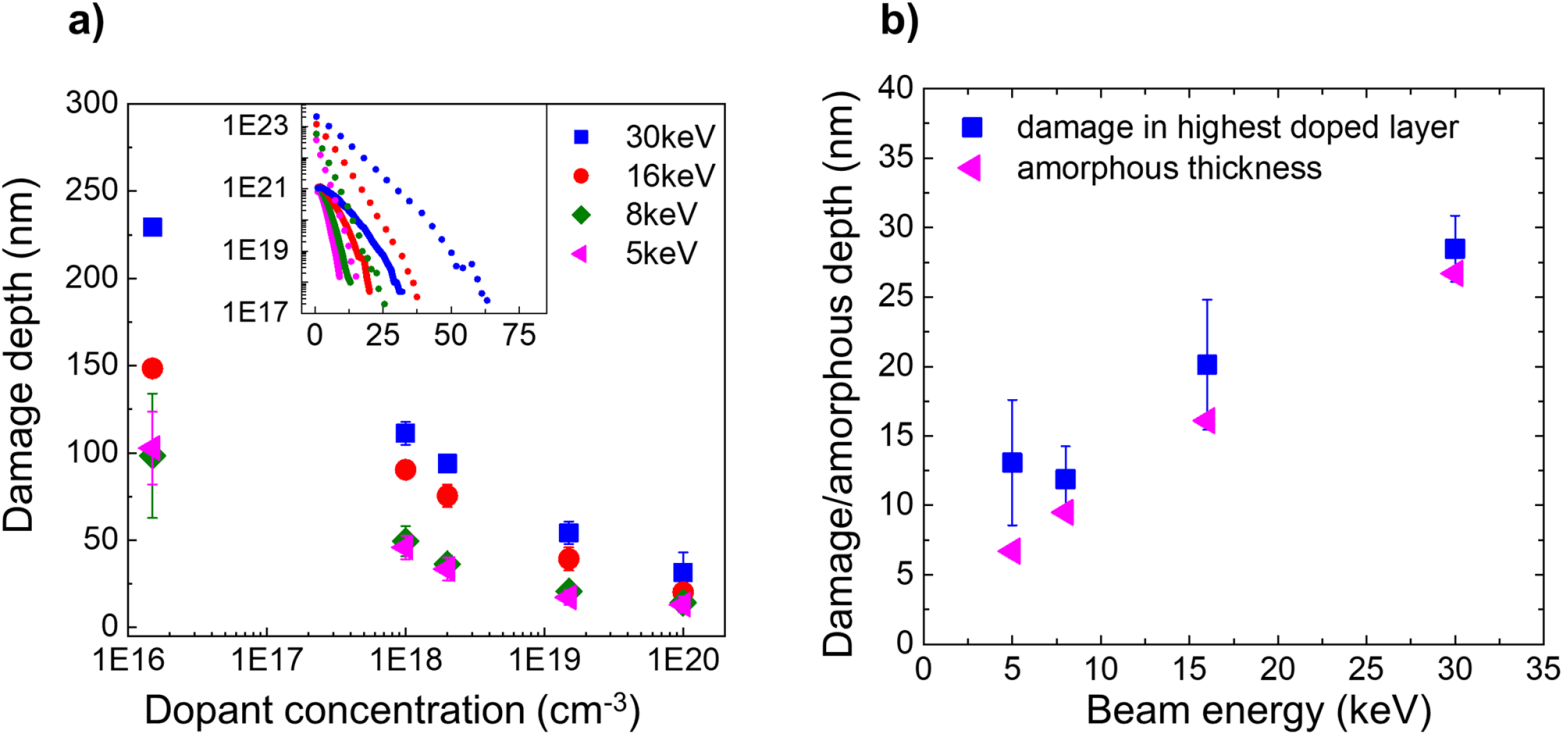
The effect of the Ga beam energy on the electrical damage depth studied using the scalpel method. (**a**) The electrical damage depths recorded for different Ga beam energies. The calculated Ga (solid lines) and vacancy concentration profiles (in cm^−3^) (dotted lines) as a function of depth (in nm) are also shown in the inset for various beam energies (coloured accordingly). (**b**) Electrical damage depths (blue squares) measured on the highest doped layers and amorphous thicknesses (pink triangles) as reported in reference^8^ for various beam energies used in the experiment.

We also studied the impact of the FIB beam energy (30 keV, 16 keV, 8 keV and 5 keV, parallel incidence) on the n-doped sample cross-section. The electrical damage depths as obtained by scalpel SSRM, are shown in Fig. 8a. The results show a strong reduction of the extent of the electrical damage when decreasing the beam energy from 30 to 8 keV. No significant difference is seen in the electrical damage depths obtained for 8 keV and 5 keV, which might be within the experimental error as the simulated vacancy profiles (see inset of Fig. 8a) only show a marginal difference when reducing the energy from 8 to 5 keV. Moreover, these results show that the electrical damage can be contained within the top 100 nm region if a beam energy less than 8 keV is used.

The simulations with SDTrimSP (version 6.01), shown in the inset of Fig. 8a, clearly show that Ga penetration profiles and vacancy distributions vary with beam energy, but they do not directly correlate to the electrical damage distribution profiles, which is in line with the results shown so far. We do, however, see that for the highest doped layer (carrier concentration of 1 × 10^20^ cm^−3^), the damage extents measured for various beam energies appear similar to the corresponding amorphous layer thicknesses as reported in the literature^8^. The comparison is shown in Fig. 8b and the good agreement indicates that the role of point defect diffusion in determining the electrical damage depth is less significant for highly doped (carrier concentrations > 10^20^ cm^−3^) layers. This is not surprising because the same amount of vacancies is injected for all the layers, meaning their impact can only be noticed if they deactivate a significant fraction of dopants, which will be determined by the ratio of injected vacancies and initial dopant concentration. In the high dopant cases the amorphization dominates the reduction of the electrical conductivity.

## Discussion

**Figure 9:**
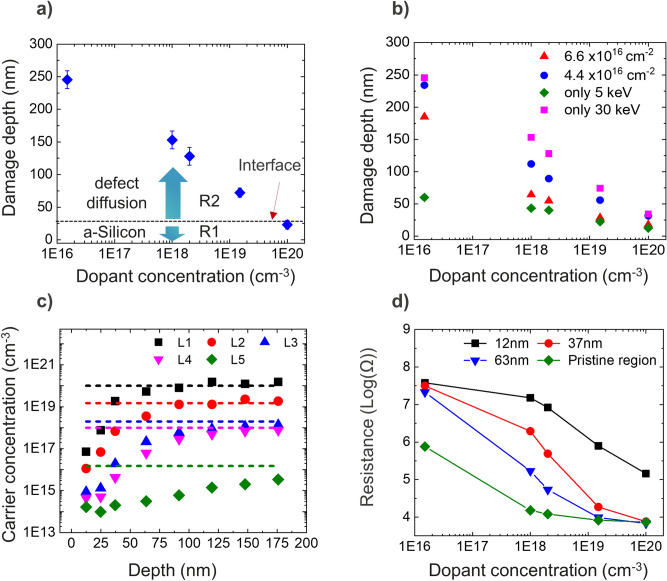
A model to describe the causes of observed damage and understanding the evaluation of existing methods for reducing the FIB damage. (**a**) Damage depth as a function of dopant concentration (when 30 keV beam is incident parallel to n-type sample cross-section) is plotted to describe the model. A high concentration of defects is injected from the interface of amorphous and crystal silicon, which then diffuse in the region R2 until they are trapped by the dopant atoms. Hence, the electrical damage depth is dependent on dopant concentration. Region R1, whose thickness is defined by the amorphous layer thickness will affect all the layers equally. (**b**) Residual electrical damage measured after low energy cleaning. The 5 keV (green diamonds) and 30 keV (pink squares) data refer to the respective stationary state. The intermediate profiles reflect the observed damage depths whereby a 30 keV treatment is followed by a 5 keV clean with limited doses. (**c**) Carrier depth profiles obtained on a FIB irradiated area using calibration values from pristine region. (**d**) Apparent calibration curves obtained from FIB processed area at various depths are shown and compared with the corresponding curve obtained on pristine area. Due to FIB damage SSRM values become higher and are more sensitive for higher doping concentrations.

Based on the presented results so far, we can now attempt to provide a comprehensive understanding of the different physical phenomena to explain the observed electrical damage depth profile. For that purpose, we can divide the electrical damage depth profiles, shown in Fig. 9a (30 keV, case Parallel, n-type), in two distinct regions. The first region (indicated as R1) represents the amorphous layer and within this region, all layers show a (similar) high resistance due to decreased mobility and/or complete dopant deactivation. In the second region (R2), beyond R1, the electrical damage extends much deeper into the Si crystal without a direct link to the Ga and vacancy distribution*.*

We established earlier that the incorporated Ga atoms do not act as active dopants and no correlation to Ga-depth distribution was found. However, we do find that both the Ga profile and the electrical damage depth depend on the angle of incidence as well as the beam energy. Both parameters determine the total amount of point defects generated in the Si. This suggests that the link to the FIB conditions must be found in the mechanisms governing the general diffusion of point defects. The process concurrent with the incorporation of the Ga atoms is the creation of collision cascades whose atomic displacements lead to the formation of vacancies and interstitials. Even beyond the amorphous layer, the injected concentration of vacancies (and interstitials) is still very high. These defects exhibit a high diffusivity at room temperature^39,40^ such that they can easily diffuse into the sample and create an exponentially decaying point defect profile. During this process they can interact with a fraction of the dopant atoms to form defect-dopant complexes leading to the deactivation of dopants^27–29^. Obviously, the effect of this interaction will only be detected when the concentration of defects is large enough such that a sizeable fraction of the dopants is deactivated*.* For the same amount of vacancies injected*,* it is clear that the effect will be visible much deeper for a lowly doped layer than for a highly doped layer. This is in line with our observations so far. The observed increase in the electrical damage depth with decreasing current density as well as the extent of Ga diffusion in Si combined with the fact that Ga diffusion in Si is controlled by vacancy mechanism^36,37^, suggests that a defect diffusion is the determining processes controlling the damage depth extent. This behaviour can be understood from the phenomenon called dynamic annealing of defects during implantation^38^. As the current density reduces, the time between the arrival of two consecutive ions within an area increases, thus providing more time for the defects to annihilate or diffuse. Now that more defects can escape from the amorphous region into the lattice, they can diffuse deeper before being completely trapped by dopant atoms. This reduction (and/or annihilation) of the point defect density in the region prone to amorphization also leads to a smaller amorphous layer thickness at a lower current density.

While investigating the details of the resistance profiles (Figs. 3a and 5b), we note that the peak resistance is not found at the surface but is located deeper in the sample. This is clearly visible in case Normal for medium (~ 10^18^ cm^−3^) and lowly doped (~ 10^16^ cm^−3^) layers, whereas for case Parallel the effect is only seen for lowly doped layers. This suggests a higher surface conduction potentially due to a higher Ga surface concentration. Indeed, in steady state (i.e. at high Ga dose, > 10^17^ cm^−2^) the Ga distribution will have a maximum at the surface and reach a level inversely proportional to the sputter yield^30^. Because of the lower sputter yield at normal incidence, more Ga ions are incorporated into the sample surface leading to a surface conduction, which is not only visible for lowly doped layers but also for medium doped layers. Whereas in case Parallel the effect can only be observed for lowly doped layers pertaining to higher sputter yield (i.e. lower Ga concentration at the surface).

## Low energy cleaning

So far, we have concluded that for normal FIB sectioning, a stationary state is achieved whereby the Ga incorporation and defect injection are counterbalanced by the effect of the material removal by the sputter process (Fig. 4). At the same time, it was shown (Fig. 8a), that milling at lower energy reduces the electrical damage depth. Unfortunately, low energy milling is more time consuming (lower current, lower sputter yield) and less precise (poorer beam focus) such that in common practice a high energy milling step is typically followed by a low energy clean. We explore the efficiency of this approach by first establishing a stationary profile with 30 keV Ga and then subsequently “cleaning” the surface with a lower energy (5 keV) beam. As it can be seen in Fig. 9b, with increasing the dose of the 5 keV clean a decrease in the overall damage depths is observed. The lowest doses of the 5 keV beam first removes the damage of the 30 keV partially, which leads to a shift of the damaged region towards the surface. Only at sufficiently high doses, all the damage will be removed, and the stationary profile related to the 5 keV bombardment will be established. Depending on the angle of incidence, the sputter yield for silicon will vary from ~ 2 (Normal) to 13 (~ 3°), implying that a dose of at least 1 × 10^17^ cm^−2^ is required to remove the fully damaged layer of the 30 keV process (which is ~ 250 nm). This approach will only be effective provided that the cleaning step completely removes the 30 keV related damage profile and establishes its own reduced damage distribution.

## Carrier quantification

So far, we have concentrated on determining the depth over which the electrical properties are affected. Since even after milling with very low FIB energies some damage remains, the quantification of the SSRM data, i.e. converting resistance to active carrier concentration, needs to be re-evaluated to account for this effect. Common practice in quantifying SSRM data is to establish a relationship between resistance and carrier concentration for each tip by measuring a known staircase calibration sample (shown in Fig. 1a,b). This calibration curve can subsequently be used to quantify the unknown sample. When applying this procedure (using the calibration values from the pristine region) to the (scalpel) resistance values obtained on a FIB irradiated area (30 keV, case Parallel) of the staircase sample (n-type), large variations as a function of depth in the apparent carrier concentration are seen even though the layers are uniformly doped. The expected values are indicated by the dashed lines in Fig. 9c whereas the actual results demonstrate an underestimation of more than 3 orders of magnitude in the near surface regions (40 nm). One could argue here that for the same number of injected defects more or even a complete deactivation is expected in the low doped layers as compared to the high doped layers. This is not clearly observed in the carrier profiles shown in Fig. 9c. However, we notice in Fig. 2c that the relative increase in the resistance is higher for the low doped layers, implying that the defects are present in excess in lower doped regions. Moreover, a complete deactivation will not result into an infinite resistance due to the presence of a conductive path provided by the electrically active defects. Consequently, the measured carrier density cannot be easily translated to the number of defects and vice versa. Therefore, during the carrier quantification of an unknown sample, one should use calibration values obtained on a calibration sample which has undergone a similar FIB preparation procedure. This can nominally resolve the errors but the dominance of the electrical damage over the spreading resistance typically leads to a calibration curve which shows much higher resistance values and a reduced sensitivity to small concentration variations in the low doped regions (Fig. 9d). The depth dependent effect of the FIB imposes serious constraints on the quantification of Scalpel SSRM, as depending on the material removed a different calibration curve is required (see Fig. 9d). The ultimate solution to avoid any of these FIB related effects, is to position the FIB based cross section ~ 200 to 300 nm before the area of interest and then progressively perform the final cross-sectioning using scalpel SSRM. In this manner, all the damage is removed, and a standard calibration curve can be used to convert the data. The drawback of this approach is a long scalpel measurement time and concurrent tip erosion. A dual-tip system using one tip dedicated to the scalpel action and one for the SSRM measurement would resolve these limitations^41^. In contrast, Prüßing et al.^42,43^ have also suggested the use of numerical simulations for SSRM quantification in the presence of various defects. Using this approach, it is possible to further investigate the underlying deactivation mechanisms and their dependence on the dopant type and on the FIB milling parameters.

Alternatively, it is worthwhile to evaluate the use of recently emerging plasma-Xe FIB in view of SSRM sample preparation. The inert nature of Xe ions will be beneficial for materials (such as III–V materials) where Ga contamination is a problem. In comparison to Ga, the smaller interaction volume of Xe ions and the availability of higher beam currents^44^ makes it a natural choice for large volume milling.

## Conclusion

We have shown that the use of FIB for device cross-sectioning significantly impacts the subsequent electrical measurements on the surface using methods such as SSRM and SCM as the energetic Ga ions drastically change the crystallinity and electronic properties of the surface. Scalpel SSRM has been employed to reveal the extent of the electrical damage induced by the FIB irradiation along the Ga implantation depth. The results clearly reveal that the damage sensed by SSRM has a dependency on the doping level of the materials and on the Ga ion beam energy. For doping levels ranging from 1 × 10^20^ to 1.5 × 10^16^ cm^−3^, the impact of the electrical damage ranges at least from 23 to 175 nm for a 30 keV Ga beam which can be reduced to 13–100 nm with an 8 keV Ga beam. Although the depth distribution and number of defects injected during the irradiation process is governed by the beam energy and beam incident angle, their in-diffusion process causes the much larger depth over which the deactivation occurs. Since the total amount of injected point-defects are independent of the dopant concentration, the defects can migrate over longer distances inside low doped layer before being trapped by a dopant atom and hence their effect is apparent much deeper in that layer. The defect in-diffusion model is further supported by the fact that an increase in current density allows less time for diffusion to happen prior to annihilation, thus leading to smaller electrical damage depths and Ga in-diffusion profiles. Any attempt to quantify SSRM data on a FIB prepared cross section needs to be based on a calibration curve created with a calibration sample that has undergone a similar FIB preparation process. Ignoring the effect of the FIB on the calibration will otherwise lead to errors as large as three orders of magnitude.
